# Between access and anxiety: the paradox of digital mental health literacy

**DOI:** 10.3389/fpsyt.2025.1691945

**Published:** 2025-10-20

**Authors:** Anithamol Babu

**Affiliations:** ^1^ School of Social Work, Marian College Kuttikkanam Autonomous, Kuttikkanam, Kerala, India; ^2^ School of Social Work, Tata Institute of Social Sciences, Guwahati, Assam, India

**Keywords:** digital mental health, over-literacy, diagnostic anxiety, algorithmic content, epistemic burden, data dependency, digital psychiatry, mental health literacy

In the contemporary landscape of digital psychiatry, mental health literacy is widely regarded as a crucial pillar of empowerment. From telepsychiatry platforms and symptom checkers to mood-tracking applications and social media testimonials, the prevailing assumption is that increased access to information enhances mental health outcomes (1). National policies, global health consortia, and educational initiatives have consistently promoted digital literacy as a strategic imperative, particularly for underserved populations and youth (2–4). The logic underpinning this optimism is both linear and persuasive: the more individuals understand mental health through digital means, the better equipped they are to identify symptoms, seek assistance, and maintain psychological well-being (5–7). However, this narrative - frequently reproduced across disciplines - obscures a growing paradox. When individuals are inundated with excessive, conflicting, or decontextualized mental health information, the outcome is not empowerment but epistemic overload and psychological distress. This article introduces the concept of digital mental health over-literacy, referring to a state wherein the volume of knowledge surpasses an individual’s ability to integrate, interpret, or emotionally process that information.

This phenomenon is particularly salient among adolescents and young adults, who are immersed in digital ecosystems saturated with psychiatric content. Platforms such as TikTok, YouTube, and Reddit feature millions of user-generated narratives describing symptoms of ADHD, autism, borderline personality disorder, depression, and anxiety (8–12). While this content democratizes access to mental health discourse and challenges traditional clinical gatekeeping, it simultaneously blurs the boundaries between diagnostic authority and personal storytelling. Adolescents frequently internalize these narratives during critical periods of identity formation, often adopting multiple diagnostic labels to make sense of their experiences (13). This dynamic gives rise to diagnostic anxiety—a persistent preoccupation with identifying latent disorders, a fear of misdiagnosis, and a compulsive engagement with symptom checklists (14, 15). Whereas traditional cyberchondria is characterized by excessive online health-related searching, this form of over-literacy is mediated by algorithmically curated, emotionally resonant content (16–18). Rather than facilitating insight, it engenders cognitive instability, diagnostic ambiguity, and emotional vulnerability.

In contrast, digital mental health over-literacy is immersive and shaped by emotionally charged, algorithmically curated content. Unlike the episodic, text-based searches typical of cyberchondria, over-literacy saturates interpretive faculties with continuous exposure. It overwhelms interpretation rather than simply increasing information. Data dependency, in contrast, refers to behavioral over-reliance on digital metrics to validate or invalidate emotional states—often emerging as a by-product of over-literacy. These phenomena share digital mediation but operate differently at cognitive and affective levels.

A further manifestation of over-literacy is the proliferation of digital self-monitoring tools that promote themselves as instruments of psychological regulation. Apps that track mood, sleep, heart rate, and stress levels are often marketed as empowering technologies that facilitate proactive mental health management (19–21). However, for individuals with pre-existing anxiety, trauma histories, or obsessive-compulsive traits, these tools frequently become mechanisms of compulsive self-surveillance (22, 23). The ritualized tracking of bodily and emotional states contributes to what may be termed a “tyranny of data,” wherein minor fluctuations are catastrophized, and deviations from perceived normative metrics are interpreted as evidence of psychological decline. Rather than fostering resilience, such technologies encourage data dependency - a reliance on external metrics for emotional validation, which erodes trust in one’s embodied experiences (24–27). In this context, the quantification of mood replaces the intuitive understanding of emotion. Patients increasingly defer to data interpretations, asking not “How do I feel?” but “What does my app say I feel?” This phenomenon is increasingly documented in studies of digital self-tracking behavior, which show a shift from embodied self-awareness to metric-driven validation (25, 27). Such epistemic displacement undermines psychological autonomy and reinforces a form of digital fragility that is antithetical to therapeutic growth.

This overexposure to digital knowledge also reshapes the clinician–patient relationship in problematic ways. A growing number of patients enter psychiatric consultations equipped with extensive digital information—ranging from diagnostic criteria and symptom taxonomies to anecdotal treatment accounts. While such knowledge can support collaborative care, it often generates epistemic tension. Therapeutic encounters risk becoming adversarial when clinical interpretations diverge from patients’ digitally acquired beliefs. Patients may interpret professional disagreement as invalidation, while clinicians may perceive digitally informed patients as resistant or misinformed. Consequently, the therapeutic alliance—a cornerstone of effective psychiatric care—is strained by epistemic mismatch. This erosion of trust reflects interpersonal dynamics and systemic shortcomings in how digital literacy is scaffolded without interpretive, cultural, or affective guidance.

Importantly, the adverse effects of digital over-literacy are not uniformly distributed. Specific populations are particularly susceptible to its psychological burdens. Adolescents, undergoing critical developmental transitions, are highly vulnerable to diagnostic identification and social comparison. Neurodivergent individuals, such as those with ADHD or autism, often encounter difficulties in filtering and prioritizing vast amounts of information, which can exacerbate cognitive overload and emotional dysregulation. Trauma survivors, predisposed to hypervigilance and intrusive cognitive patterns, may engage in compulsive monitoring, interpreting ambiguous signals as indicators of psychological deterioration. Digital over-literacy functions as a stratified epistemic burden for these populations, compounding existing vulnerabilities rather than mitigating them. Failure to recognize this intersectional dimension risks replicating the exclusions psychiatry aims to dismantle.

At the heart of this issue is psychiatry’s enduring epistemological commitment to linearity—the belief that increased literacy directly translates into better clinical outcomes. While this assumption may hold in domains such as physical health or academic learning, it falters within the domain of mental health, where uncertainty, cultural variability, stigma, and affective complexity predominate. Excess information may not facilitate clarity in such contexts but instead generate confusion, fear, and identity destabilization. Digital over-literacy, therefore, is not merely a benign by-product of accessibility; it constitutes a significant risk factor for emotional distress, diagnostic overreach, and therapeutic disengagement.

To address this emerging challenge, psychiatry must reconceptualize digital mental health literacy as a bounded, context-sensitive, and critically mediated competency. First, it is imperative to promote critical literacy—evaluating source credibility, recognizing algorithmic biases, and distinguishing between anecdotal narratives and clinically verified information. Users must be equipped to access digital content and interrogate its epistemic foundations and emotional implications. Second, literacy interventions must be culturally contextualized. Diagnostic categories such as depression, trauma, or anxiety do not carry uniform meaning across different sociocultural and linguistic settings. Effective interventions must therefore engage with local idioms of distress, indigenous knowledge systems, and community-based explanatory models. The imposition of Western psychiatric taxonomies through digital media risks entrenching epistemic colonialism. This critique aligns with scholarship in global mental health that challenges the epistemic dominance of Western psychiatric norms. Without grounding in local idioms of distress, digital psychiatric frameworks risk marginalizing culturally rooted understandings of suffering (28). Deepening engagement with such frameworks is essential to avoid replicating historical exclusions in a digital form. Third, psychiatry must articulate the concept of bounded literacy - recognizing that there are thresholds beyond which further searching, self-monitoring, or knowledge accumulation may become psychologically counterproductive. For instance, thresholds might include excessive time spent on symptom-checking platforms, emotional distress triggered by minor biometric deviations, or compulsive engagement with multiple mental health communities without clinical follow-up. Bounded literacy encourages clinicians and users alike to recognize signs of informational saturation, and to view pausing or disengaging as a legitimate act of self-care. Patients need guidance on when to pause, trust their embodied awareness, and disengage from potentially destabilizing informational loops. These arguments have significant implications for mental health policy, clinical practice, and research. Policymakers must move beyond quantitative metrics such as downloads or engagement rates and instead evaluate the qualitative impact of digital interventions—particularly their effects on emotional resilience, clinical trust, and psychological safety. Alternative indicators for evaluating digital interventions could include user-reported shifts in help-seeking behavior, self-reported trust in mental health professionals’ post-intervention, or qualitative feedback on emotional safety and cultural fit. Clinicians should screen for signs of digital over-literacy during assessments, including compulsive app use, diagnostic self- labeling, and epistemic scepticism toward professional guidance. Signs of digital over-literacy may include compulsive engagement with mental health apps, frequent self-diagnosis using online symptom checklists, emotional distress triggered by app feedback, and resistance to clinical reframing due to digital preconceptions. Clinicians should also be trained in strategies to address epistemic dissonance with empathy and humility. For policymakers, alternative impact indicators could include user-reported shifts in help-seeking behavior, self-reported trust in mental health professionals’ post-intervention, or qualitative feedback on emotional safety and cultural fit. Moving beyond simplistic metrics like app downloads and engagement rates, these nuanced indicators can more accurately capture the real-world psychological impact of digital mental health tools.

From a research perspective, there is a pressing need for longitudinal, mixed-methods, and intersectional studies to identify the thresholds at which digital literacy becomes detrimental. Empirical instruments should be developed to capture digital over-literacy as a distinct psychosocial construct and to trace its interactions with identity, cognition, and cultural context.

Digital mental health literacy remains an essential component of contemporary psychiatric practice. However, the claims advanced in this article are necessarily limited by the nature of opinion-based reflection and do not rest on empirical generalizability. While the observations presented here are informed by interdisciplinary insight and emerging clinical concerns, they should be regarded as heuristic and provisional. Rigorous empirical research is required to substantiate, refine, or challenge the construct of digital over-literacy, particularly across culturally diverse clinical contexts. Future investigations should examine how platform design, algorithmic logic, identity formation, and cognitive vulnerability influence users’ engagement with mental health content. Such research will be instrumental in guiding the ethical design of digital interventions that are not only accessible but also cognitively sustainable and affectively safe. Until this evidence base is developed, psychiatry must approach digital literacy with ambition and caution—committed to expanding access and cultivating epistemic discernment and emotional containment. Ultimately, while digital tools can democratize access to mental health knowledge, they also demand critical mediation. Over-literacy is not a failure of access, but a failure of curation, containment, and care. As psychiatry enters the algorithmic era, the challenge is informing, holding, and guiding.
